# Erratum

**DOI:** 10.1002/acn3.51600

**Published:** 2022-06-06

In the article by Carrasco‐Rozas et al.,^1^ the affiliation 1 was incorrect and this has been corrected as follows.

From:

Neuromuscular Disorders Unit, Neurology Department, Hospital de la Santa Creu i Sant Pau, Universitat Autònoma de Barcelona, Barcelona, Spain.

To:

Neuromuscular Disorders Unit, Neurology Department, Hospital de la Santa Creu i Sant Pau and Biomedical Research Institute Sant Pau (IIB Sant Pau). Departament de Medicina, Universitat Autònoma de Barcelona, Spain.
